# Installing a Single Monomer within Acrylic Polymers Using Photoredox Catalysis

**DOI:** 10.1021/jacs.3c12221

**Published:** 2023-12-21

**Authors:** Jared G. Baker, Richard Zhang, C. Adrian Figg

**Affiliations:** Department of Chemistry and Macromolecules Innovation Institute, Virginia Tech, Blacksburg, Virginia 24061, United States

## Abstract

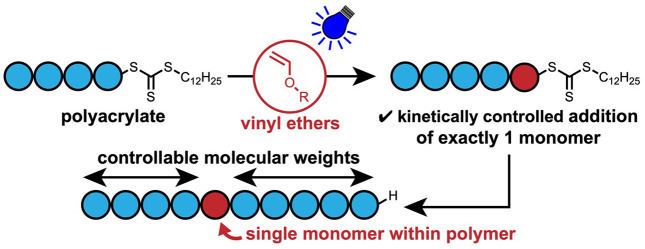

Incorporating exactly one monomer at a defined position during a chain polymerization is exceptionally challenging due to the statistical nature of monomer addition. Herein, photoinduced electron/energy transfer (PET) enables the incorporation of exactly one vinyl ether into polyacrylates synthesized via reversible addition–fragmentation chain transfer (RAFT) polymerization. Near-quantitative addition (>96%) of a single vinyl ether is achieved while retaining >99% of the thiocarbonylthio chain ends. Kinetic studies reveal that performing the reactions at 2 °C limits unwanted chain breaking events. Finally, the syntheses of diblock copolymers are reported where molecular weights and dispersities are well-controlled on either side of the vinyl ether. Overall, this report introduces an approach to access acrylic copolymers containing exactly one chemical handle at a defined position, enabling novel macromolecular architectures to probe structure–function properties, introduce sites for de/reconstruction, store information, etc.

Functionality plays a critical role in polymer properties.^1−4^ For example, routes to synthesize sequence-defined and sequence-controlled polymers are pursued to mimic the complexity of and information stored in biomacromolecules (e.g., proteins, DNA, polysaccharides).^5−10^ However, there are very few examples demonstrating the synthesis of polymers with defined functional group placement using chain polymerization techniques.^11−19^ In these examples, the functional group placement is either preprogrammed into the monomers, or only oligomers can be synthesized (Figure 1). This lack of examples is surprising given that the position of just a single moiety can have substantial impacts on properties. For example, a single ionic charge placed at different positions within an amphiphilic polypeptoid affected assembly properties.^20^ This observation suggests that the analogous modulation of acrylic polymer properties could be tuned via the incorporation of single functionalities. However, incorporating a single monomer within a polymer chain at a defined position is challenging because the inherent statistical growth process of common chain polymerization techniques must be overcome.^21^

**Figure 1:**
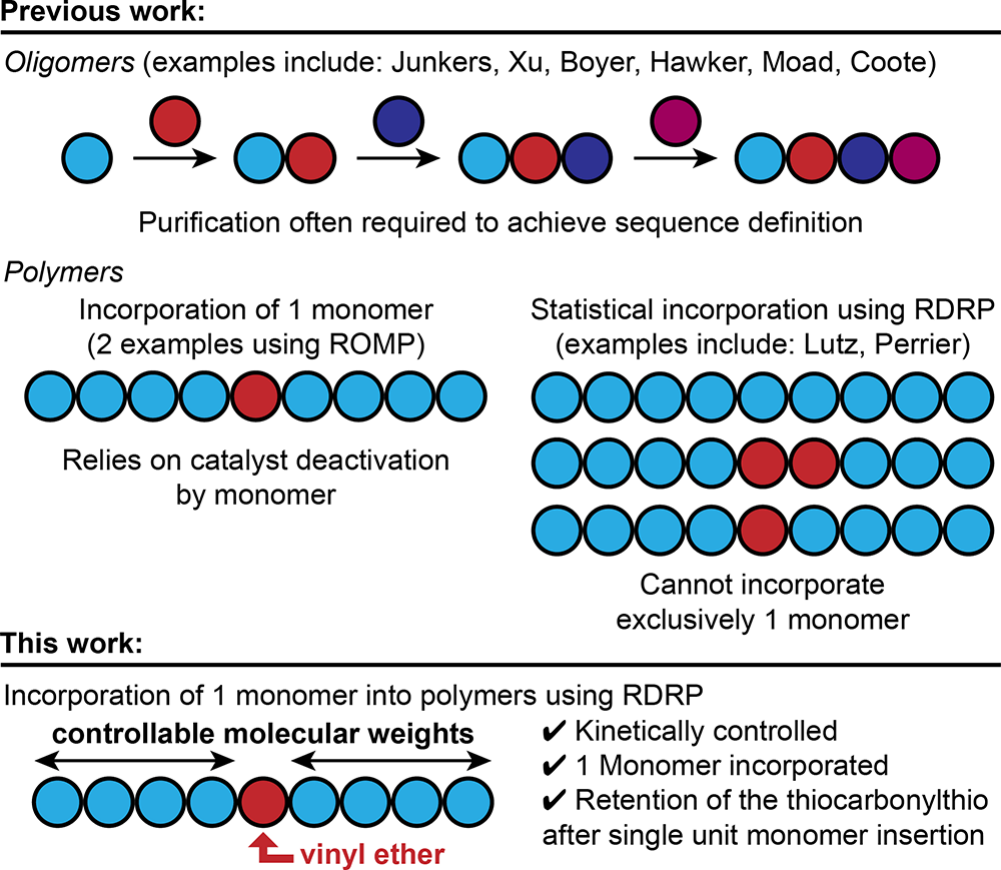
Previous work incorporating successive single-unit monomer insertion reactions to access oligomers,^11,12,15,16^ exactly 1 monomer into polymers by ring-opening metathesis polymerization (ROMP),^22,23^ and statistically incorporating monomers into polymers using reversible–deactivation radical polymerization (RDRP)^24,25,28^ versus the incorporation of exactly 1 vinyl ether monomer into polyacrylates using RDRP.

Monomer design can be used to place single functionalities in polynorbornenes using ring-opening metathesis polymerization (ROMP, Figure 1).^22,23^ However, accessing analogous structures using reversible-deactivation radical polymerization (RDRP) has not been accomplished because radical polymerizations rely entirely upon monomer addition kinetics. A chain-end deactivation approach similar to the one used for ROMP is inaccessible due to the radical nature of RDRP. Notable examples approaching this goal were pioneered by Lutz leveraging the poor homopolymerizability of maleic anhydride and maleimides.^24−26^ However, these monomers undergo minor homopolymerization, so defects of 0 and 2 additions exist in the polymer products, limiting access to precise macromolecular structures.^27−29^ There are no examples that use RDRP to insert exactly 1 monomer at a defined position in a macromolecule because previous strategies have relied upon monomer addition kinetics without sufficient selectivity. Kinetic selectivity over monomer addition is paramount because defects of 0 and 2 additions cannot be purified out of polymer samples, a distinct challenge compared to oligomeric single-unit monomer insertion (SUMI) approaches.^11,12,15,16^ Therefore, delineating the effects of precise monomer stoichiometry and position on properties (e.g., solution/bulk assembly, antimicrobials, information storage) would be convoluted by the defects in functionalities.

Herein, the slow radical polymerization kinetics of vinyl ethers^30^ are used to control the insertion of exactly one monomer within an acrylic polymer (Figure 1). Vinyl ethers are usually only used in radical polymerizations as comonomers or to switch polymerization mechanisms.^30−32^ The Barner-Kowollik lab used vinyl ethers to end-cap polymers synthesized via reversible addition–fragmentation chain transfer (RAFT) polymerization.^33^ However, multiple additions occurred, and substantial amounts of the thiocarbonylthio (TCT) chain end were lost due to irreversible termination. Even if single additions and minimal loss of the TCT had been achieved, chain extensions from vinyl ethers (less-activated monomers) with acrylates (more-activated monomers) are unfavorable and contradict blocking order requirements of traditional RAFT polymerization.^34^

We hypothesized that photoinduced electron/energy transfer (PET) reactions^35^ can be used to insert a single vinyl ether within a polyacrylate. PET-mediated radical introduction leads to fewer termination events and more uniform polymer end groups than other initiation techniques.^36,37^ We anticipated that these qualities would lead to the retention of the TCT during the SUMI reaction. If successful, chain extension of the TCT-terminated polymer containing a single vinyl ether could be possible adapting a recent photomediated approach to flip blocking order.^38^ This process would result in the first synthetic method to access acrylic copolymers with defined lengths and low dispersities on either side of exactly 1 monomer, a critical first step toward incorporating defined functional group sequences within polymers.

Poly(methyl acrylate) (PMA, Figure S1) synthesized using PET-RAFT conditions and terminated with a trithiocarbonate was used to study the SUMI reaction with benzyl vinyl ether (BVE) (Figure 2A). Three different initiation methods were screened: thermal at 75 °C,^39^ photoiniferter at 25 °C with 365 nm light,^40,41^ and PET using *fac*-Ir(ppy)_3_ at 25 °C with 455 nm light.^36^ The thermal activation method led to vinyl ether oligomerization and termination (Figure S2). Photoactivation pathways led to significant incorporation of BVE by ^1^H NMR spectroscopy analysis (97% for photoiniferter, Figure 2B, **a**; 91% for PET, Figure 2C, **a**). While the photoiniferter method resulted in near-quantitative BVE incorporation, ^1^H NMR spectroscopy indicated that ∼20% of the TCT was lost during the reaction (Figure 2B, **c**), analogous to reported results.^33^ Conversely, the PET initiation pathway led to minimal loss of the TCT (4%, Figure 2C, **c**).^36^ However, longer reaction times at 25 °C did not improve BVE incorporation (Figure S3).

**Figure 2:**
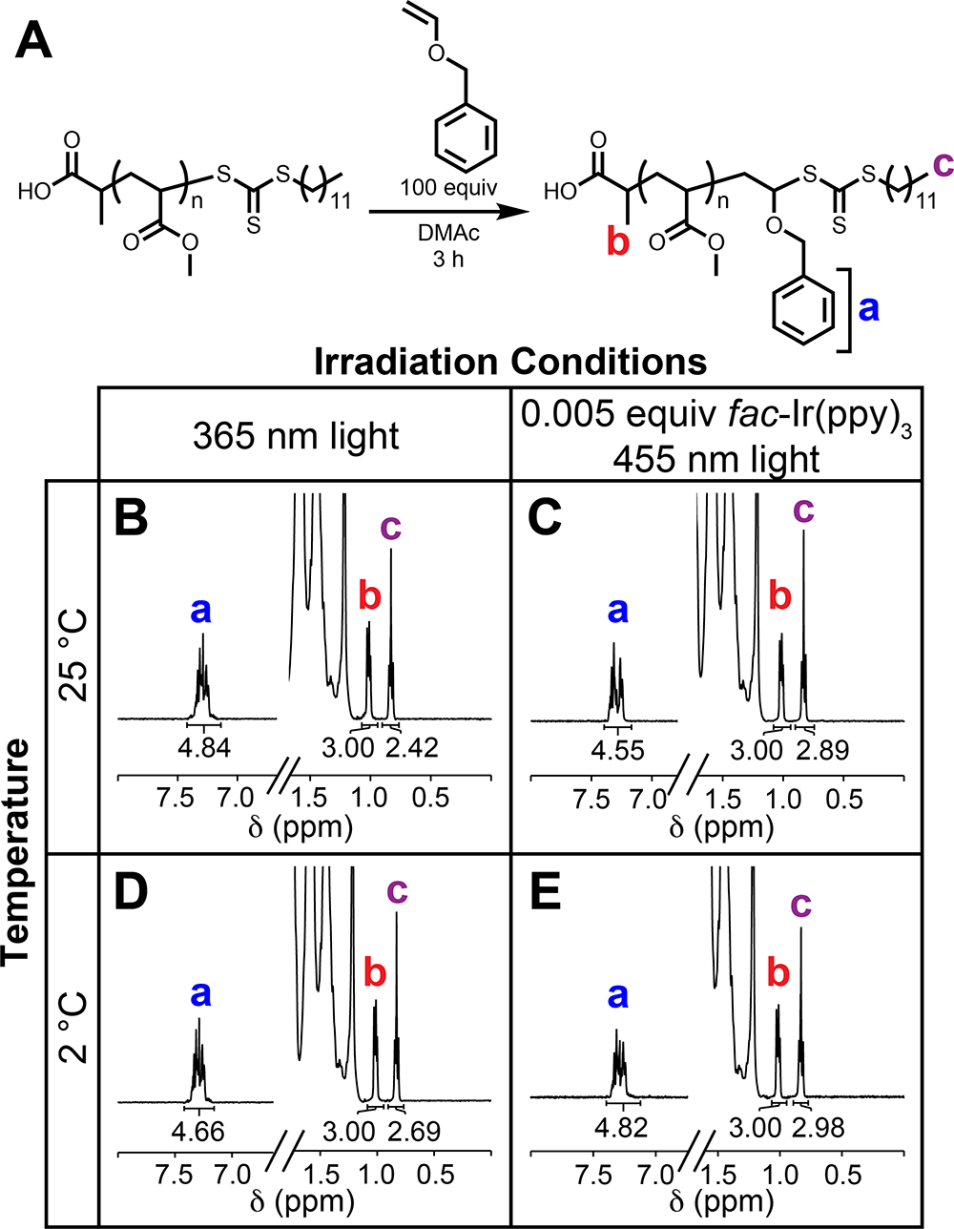
Scheme of the single-unit monomer insertion reaction (A), ^1^H NMR spectrum of PMA-BVE at 25 °C using 365 nm light (B), 25 °C using 455 nm light (C), 2 °C using 365 nm light (D), and 2 °C using 455 nm light (E).

We hypothesized that lower reaction temperatures would lead to high BVE incorporation before a significant loss of TCT occurs. We expected that TCT loss was primarily occurring by termination and chain breaking events (Figure S4), so cooling down the temperature would slow down these processes and result in higher retention of the TCT.^42^ At 2 °C, retention of the TCT was improved using either photoactivation approach. However, PET activation led to >99% retention of TCT and >96% BVE incorporation by ^1^H NMR spectroscopy (Figure 2E, **a** and **c**; Figure S5) compared to 90% retention of TCT and 93% BVE incorporation using a photoiniferter pathway (Figure 2D, **a** and **c**).

Trapping studies were conducted to probe if 2 °C slowed down rates of TCT activation by both PET and chain transfer during the SUMI reaction (Figure 3A).^43−45^ We hypothesized that the TCT rate of activation by the PET catalyst (*k*_A, PET_) would not be as affected by the temperature compared to the TCT activation by chain transfer (*k*_A, CT_).^46,47^ Importantly, these experiments solely measured the rate of TCT consumption via either radical quenching or monomer addition; they did not measure the rates of degenerative chain transfer characteristic of RAFT processes.

**Figure 3:**
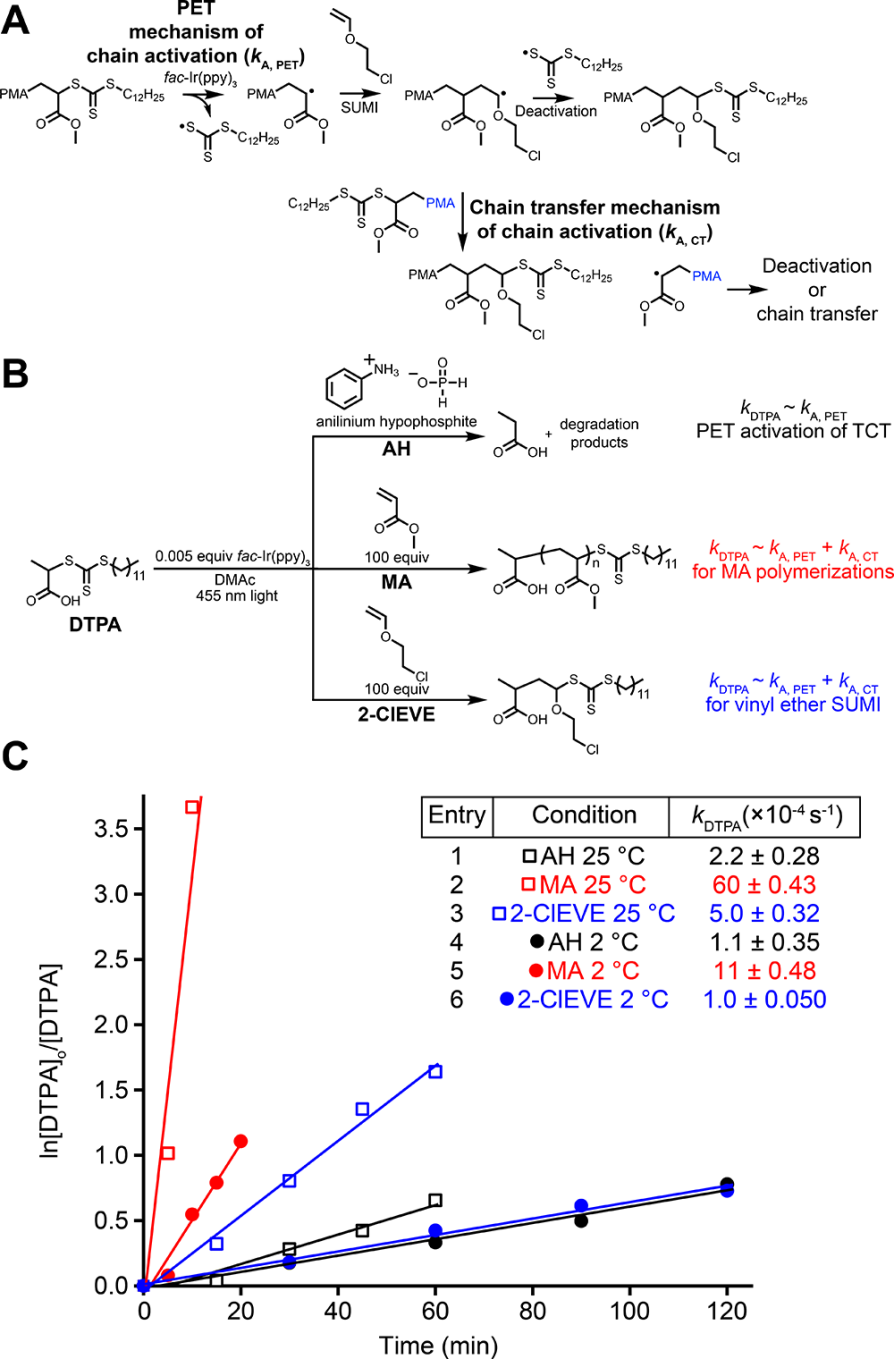
(A) Scheme describing chain activation via PET catalysis and chain transfer; (B) Schemes of different conditions to study the consumption of 2-(dodecylthiocarbonothioylthio) propionic acid (DTPA); (C) Kinetic data for the consumption of DTPA with methyl acrylate (MA), 2-chloroethyl vinyl ether (2-ClEVE), or anilinium hypophosphite (AH). All experiments monitored the disappearance of the methine proton of DTPA at δ = 4.68 ppm via ^1^H NMR spectroscopy.

The disappearance of the methine proton adjacent to the TCT (δ = 4.68 ppm) of 2-(dodecylthiocarbonothioylthio) propionic acid (DTPA) was monitored to extract an apparent rate constant of DTPA consumption (*k*_DTPA_, Figure 3B).^48^ The rate of consumption of DTPA under different conditions was then used to identify the proportion of *k*_A, PET_ versus *k*_A, CT_ occurring during the addition of a single vinyl ether.

Anilinium hypophosphite (AH), a radical-scavenging hydrogen atom source,^48^ was used in 15-fold excess to prevent TCT activation via chain transfer (Figure S6). The *fac*-Ir(ppy_3_) and DTPA concentrations were kept constant throughout the different conditions. Resultantly, these rates represent radical introduction and consumption of DTPA when all other possible chain transfer and monomer addition events are eliminated. Therefore, the *k*_DTPA_ values measured in the presence of AH at 25 °C (Figure 3C, entry 1) and 2 °C (Figure 3C, entry 4) are directly proportional to *k*_A, PET_. Since radical concentration is assumed to be constant, increases in *k*_DTPA_ under different conditions will provide insight into the relative rates of *k*_A, PET_ versus *k*_A, CT_. An increased rate in *k*_DTPA_ means that DTPA is activated by chain transfer in addition to PET.

The *k*_DTPA_ in the presence of AH decreased from 2.2 × 10^–4^ s^–1^ at 25 °C to 1.1 × 10^–4^ s^–1^ at 2 °C, indicating that *k*_A, PET_ is slowed down by 50% at 2 °C compared to 25 °C. This result indicates that lowering the temperature decreases the overall number of radicals introduced into the system via PET, leading to fewer termination events.

The *k*_DTPA_ in the presence of MA decreased from 60 × 10^–4^ s^–1^ at 25 °C (Figure 3C, entry 2) to 11 × 10^–4^ s^–1^ at 2 °C (Figure 3C, entry 5; Figure S7). The decrease in rate suggests that *k*_A, CT_ is more affected by temperature than *k*_A, PET_. At 25 °C, the difference in rate between AH and MA increases by 30×, while the difference in rate between the two conditions at 2 °C only increases by 10×. Additionally, DTPA is consumed significantly faster in the presence of MA. Therefore, activation by chain transfer (*k*_A, CT_) is the predominate mode of DTPA consumption in the presence of monomers that homopolymerize because *k*_DTPA_ is significantly faster than *k*_A, PET_. These results confirm that conventional PET-RAFT activation of TCTs occurs predominately via degenerative chain transfer, instead of PET.

The *k*_DTPA_ in the presence of 2-ClEVE decreased from 5.0 × 10^–4^ s^–1^ at 25 °C (Figure 3C, entry 3) to 1.0 × 10^–4^ s^–1^ at 2 °C (Figure 3C, entry 6; Figure S8). These results show that (1) DTPA consumption in the presence of vinyl ethers decreases with temperature and (2) at 2 °C, the *k*_DTPA_ is the same value as *k*_DTPA_ in the presence of AH. Therefore, we concluded that chain transfer does not play a significant role in DTPA consumption in the presence of vinyl ethers. Furthermore, these observations suggested that chain breaking events were also significantly slowed down at the lower temperatures.

Next, we sought to confirm that (1) exactly one vinyl ether was added to each chain and (2) the conditions were amenable to other vinyl ethers. SUMI reactions were performed with five vinyl ethers: BVE, 2-ClEVE, *n*-butyl vinyl ether (*n*-BuVE), *iso*-butyl vinyl ether (*iso*-BuVE), and di(ethylene glycol) vinyl ether (DEGVE) and analyzed using matrix assisted laser desorption/ionization time-of-flight mass spectrometry (MALDI-TOF MS) (Figure 4 and Figures S9–S14).

**Figure 4:**
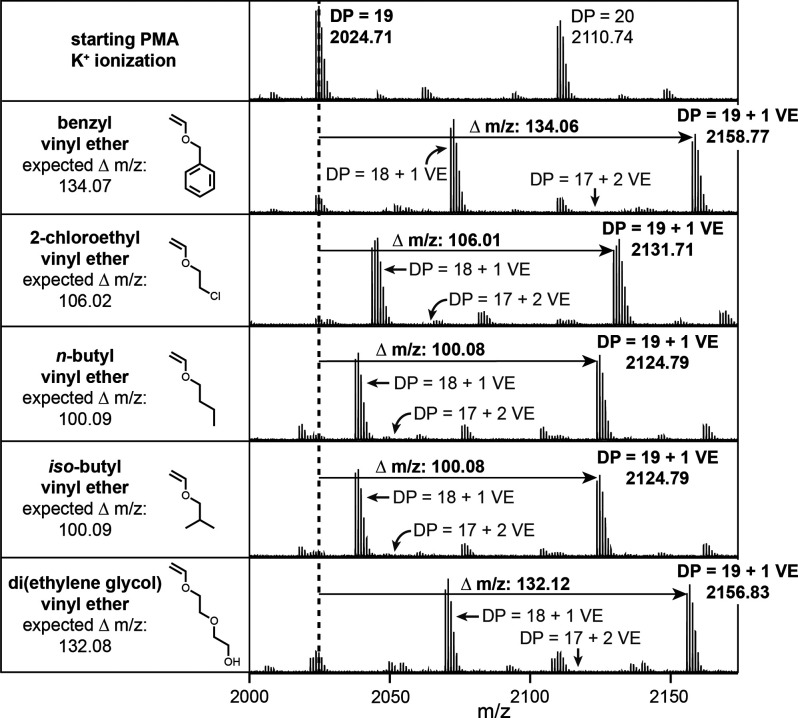
MALDI-TOF MS spectra of 5 vinyl ethers (BVE, 2-ClEVE, *n*-BuVE, *iso*-BuVE, and DEGVE) added to poly(methyl acrylate) (PMA).

The expected *m*/*z* shift of each vinyl ether was observed. For BVE, a shift of 134.07 *m*/*z* was expected and a shift of 134.06 *m*/*z* was observed. No double addition peaks of BVE were observed. The main potassium adduct peak corresponds to PMA-BVE with a TCT on the chain end. Analogous observations of single addition, retention of TCT, and minimal starting PMA were observed for 2-ClEVE, *n*-BuVE, *iso*-BuVE, and DEGVE.

Size-exclusion chromatography (SEC) analysis was used to confirm that no vinyl ether polymerization occurred. No shift in retention time was observed, indicating no vinyl ether polymerization (Figure 5). A minor distribution at lower retention times following the SUMI reaction potentially results from chain breaking events (Figure S4), leading to polymer–polymer coupling of the resultant pendent or backbone radical with another polymer. This distribution is double the molecular weight of the starting PMA (Figure S15) and accounts for ∼4 mol % of the total population. Considering the ^1^H NMR spectra, MALDI-TOF MS spectra, and SEC chromatograms, exactly one vinyl ether is added to each chain end.

**Figure 5:**
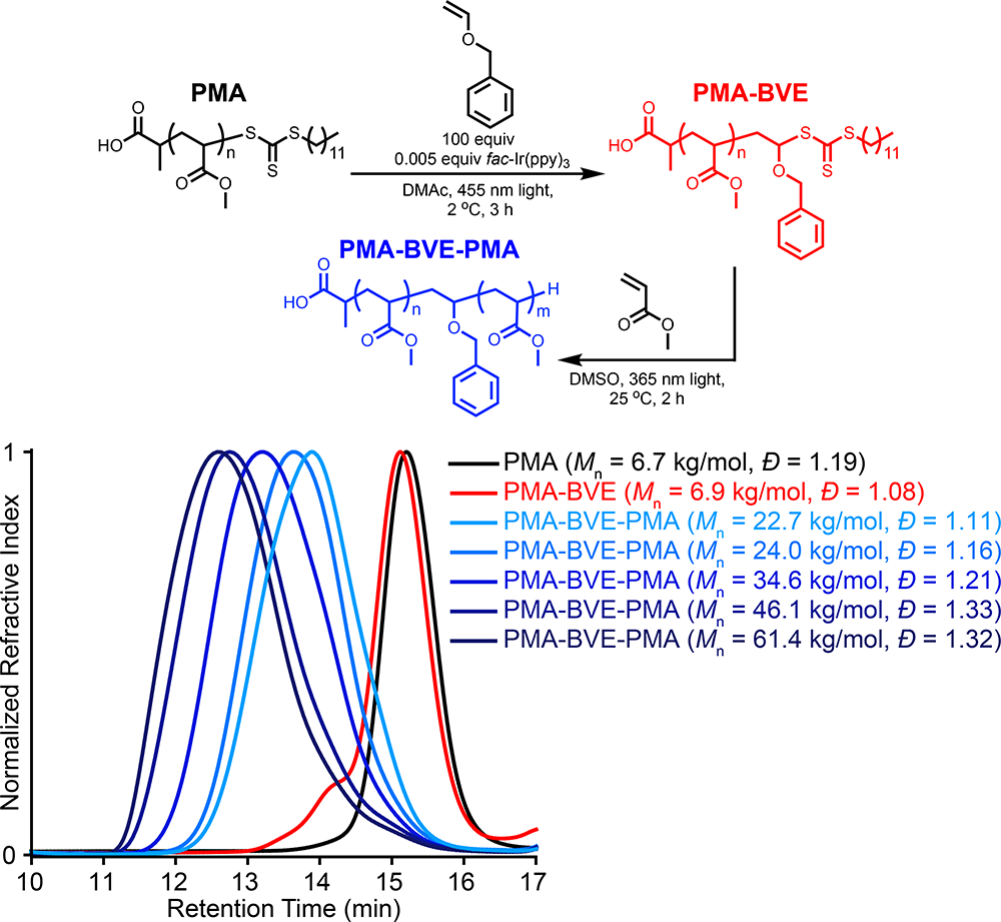
Scheme and size-exclusion chromatograms of the insertion of BVE into a PMA chain and subsequent chain extensions from poly(methyl acrylate)-benzyl vinyl ether (PMA-BVE) using methyl acrylate to achieve PMA-BVE-PMA.

Chain extensions with methyl acrylate (MA) were performed to confirm TCT retention on the chain end (Figure 5, Figure S16). Photoiniferter conditions were used, as PET-RAFT conditions led to multimodal traces (Figure S17).^38^ Polymerizations were set up targeting different molecular weights (Figure 5). Uniform shifts to higher molecular weights of 22.7, 24.0, 34.6, 46.1, and 61.4 kg/mol were observed when 100, 200, 300, 400, and 500 equiv of MA were used, respectively (Figure 5 and Figures S16, S18–21). However, loss of the TCT occurred during this step, indicated by a loss of absorbance at the TCT absorption at 365 nm (Figure S22). Importantly, these data demonstrate that the molecular weight of the polymer following the SUMI reaction can be controlled.

In conclusion, photochemistry enabled the placement of exactly one vinyl ether within the acrylic polymers. These results are a significant step toward sequence-defined polymers and new polymer architectures. We expect that these architectures will play a critical role in (1) probing structure–function properties, (2) incorporating sites for controllable polymer deconstruction and reconstruction, and (3) accessing macromolecular architectures where the placement of defined chemical moieties dictate function (e.g., polymer–protein conjugates, ionic polymers, antimicrobial polymers, information-storage polymers, etc.).

## Supporting Information Available

Additional ^1^H NMR spectra, size exclusion chromatograms, mass spectra, and experimental details, materials, and methods. (PDF)
