# Supplementary material for: Installing a Single Monomer within Acrylic Polymers Using Photoredox Catalysis
Source: J Am Chem Soc. 2023 Dec 21;146(1):106–11. doi: 10.1021/jacs.3c12221 (PMC10785814; doi:10.1021/jacs.3c12221)
Supplement: Supplementary file 1 — ja3c12221_si_001.pdf [file ja3c12221_si_001.pdf]

Supplementary Information for

Installing a Single Monomer within Acrylic Polymers using Photoredox Catalysis

Jared G. Baker, Richard Zhang, and C. Adrian Figg*

Department of Chemistry and Macromolecules Innovation Institute, Virginia Tech, Blacksburg, Virginia 24061, United States

Experimental

Materials

Anilinium hypophosphite (Sigma-Aldrich, 97%) was used as received. Methyl acrylate (MA, Thermo Scientific, 99%), benzyl vinyl ether (BzVE, Synthonix, 97%), 2-chloroethyl vinyl ether (2-ClEVE, Acros Organics, 95%), *n*-butyl vinyl ether (*n*BuVE, Alfa Aesar, 98%), isobutyl vinyl ether (*iso*BuVE, TCI, 99%), and diethylene glycol vinyl ether (DiEGVE, TCI, 96%) were filtered through basic alumina prior to use. *fac*-Ir(ppy)$_3$ (Strem Chemicals, 95%) was prepared as a 10 mg/mL solution in *N,N*-dimethylacetamide (DMAc) prior to use. 2-(Dodecylthiocarbonothioylthio)propionic acid (DTPA) was adapted and synthesized from a previous report.[1] All solvents were used as received.

For a visible light source, 76.2 cm Supernight Blue or Green LED Light Strips were purchased from Amazon and placed on a Xnrtop Silver Tone Aluminum Radiator Heatsink Heat Sink 150 × 80 × 27 mm from Amazon. For an ultraviolet light source, an Everbeam 365 nm 50 W UV LED Black Light from Amazon was used.

Characterization

^{1}H NMR spectroscopy was conducted on either an Agilent U4-DD2 400 MHz or a Bruker Avance II 500 MHz at 25 °C. DMSO-*d*$_6$ (Cambridge Isotopes Laboratories, Inc., 99.9%) and $CDCl_3$ (Cambridge Isotopes Laboratories, Inc., 99.8%) were used as received.

Size Exclusion Chromatography (SEC) was performed in *N*,*N*-dimethylacetamide (DMAc) with 50 mM LiCl at 50 °C at a flow rate of 0.5 mL min^{-1} (Agilent isocratic pump, degasser, and autosampler, columns: TOSOH TSKgel Guard Alpha and TOSOH TSKgel Alpha-3000: molecular weight range 0–1 × 10^5 g mol^{-1}). Detection consisted of a Wyatt Optilab refractive index detector operating at 785 nm, a Wyatt DAWN multi-angle light scattering detector operating at 783 nm, and an Agilent MWD operating at 365 nm. Absolute molecular weights and dispersities were calculated with the Wyatt ASTRA software and off-line d*n*/d*c* analysis.

Visible light intensity was measured with an International Light Technologies ILT-350 illuminance spectrophotometer with a NIST traceable ISO17025 accredited calibration.

Matrix-assisted laser desorption/ionization time-of-flight mass spectrometry (MALDI-TOF MS) was performed on a Bruker timsTOF fleX MALDI-2 instrument. Analysis of poly(methyl acrylate) and SUMI reaction products was performed by mixing *trans*-2-[3-(4-*tert*-

butylphenyl)-2-methyl-2-propenylidene]malonitrile (DCTB) matrix (10 mg/mL in THF), polymer solution (1.0 mg/mL in THF), and potassium trifluoroacetic acid (1.0 mg/mL in THF) at a v:v:v ratio of 5:2:2 matrix:polymer:salt, and 3 μL were spotted on a stainless steel Bruker MTP 384 target polished steel BC plate and air dried.

Procedures

Example polymerization for starting poly(methyl acrylate)

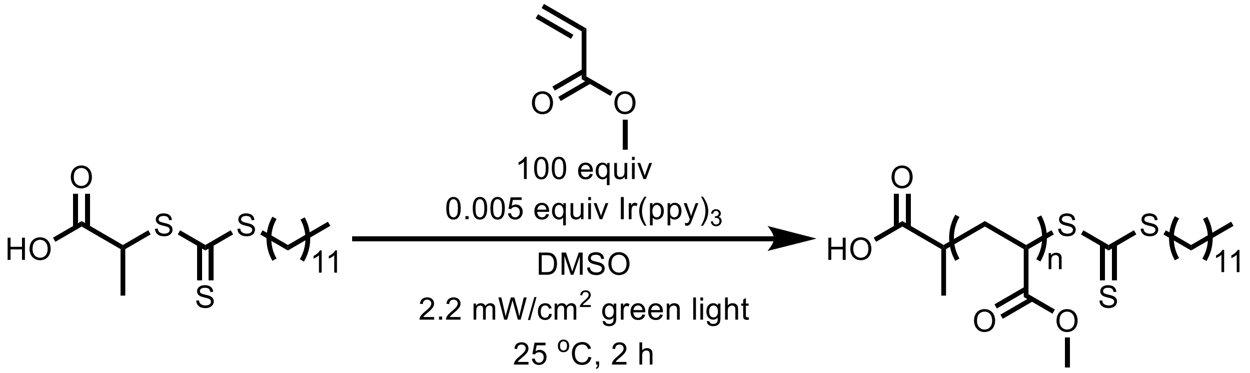

DMAc (33.6 mL), MA (18.4 g, 214 mmol), 2-(Dodecylthiocarbonothioylthio)propionic acid (750 mg, 2.14 mmol), and *fac*-Ir(ppy)$_3$ (7.01 mg, 1.07 × 10^{-2} mmol) were combined in a 50 mL Schlenk flask. The mixture was covered in aluminum foil and degassed with Argon for 30 min. The mixture was irradiated with a green LED strip (2.2 mW/cm^2) for 2 h at room temperature until achieving a conversion of 85% by ^{1}H NMR spectroscopy. The reaction mixture was then precipitated into an excess of chilled methanol 2×, and then dried under vacuum and characterized by ^{1}H NMR spectroscopy and SEC.

Example procedure for trapping studies

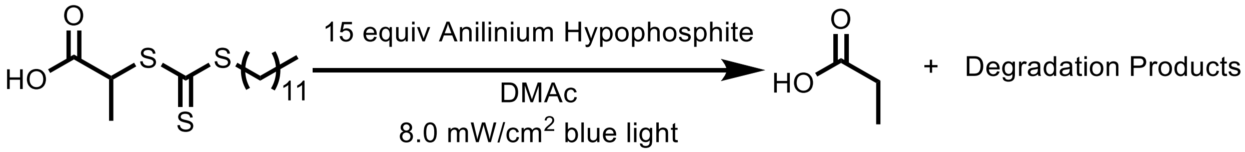

Anilinium Hypophosphite (71.6 mg, 4.50 × 10^{-1} mmol), 2-(Dodecylthiocarbonothioylthio)propionic acid (10.5 mg, 3.00 × 10^{-2} mmol), *fac*-Ir(ppy)$_3$ (9.83 × 10^{-2} mg, 1.50 × 10^{-4} mmol) were dissolved in DMAc (3.00 mL) with DMF as an internal standard in 2-dram vials and capped with rubber septum. After degassing the solution for 5 min with Argon, the vials were placed in the 2 °C fridge and equilibrated for 1 h before turning the blue LED strip on (8.0 mW/cm^2). Vials were removed at 30 min, 60 min, 90 min, and 120 min and quenched by opening the vial to air. Conversion was determined by ^{1}H NMR spectroscopy using DMSO-d_6 as a solvent and tracking the disappearance of the methine peak at $\delta = 4.64$ ppm.

Example procedure for vinyl ether small molecule analog studies

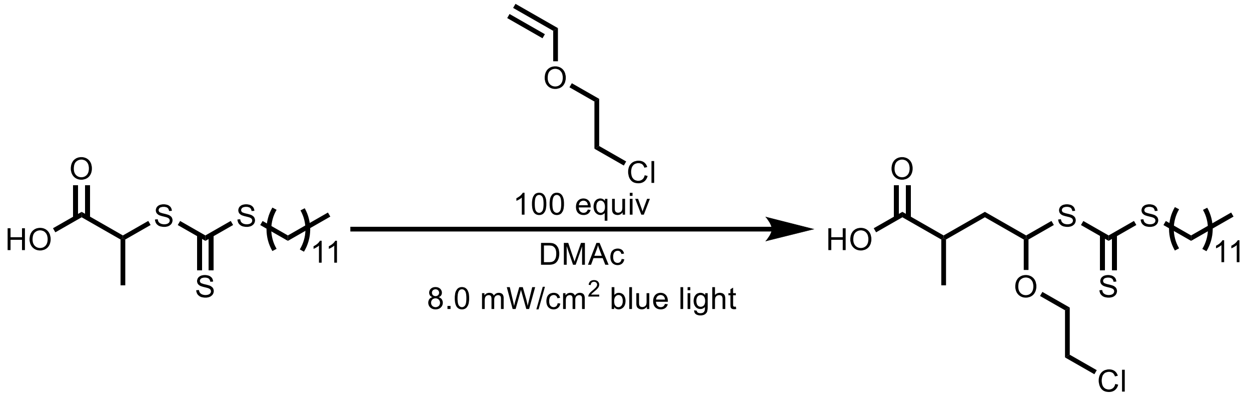

2-chloroethyl vinyl ether (1.26 g, 11.9 mmol), 2-(Dodecylthiocarbonothioylthio)propionic acid (41.6 mg, 1.19×10^{-1} mmol), *fac*-Ir(ppy)$_3$ (3.88×10^{-1} mg, 5.93×10^{-4} mmol) were dissolved in DMAc (1.46 mL) with DMF as an internal standard in 2-dram vials and capped with rubber septum. After degassing the solution for 5 min with Argon, the vials were placed in the 2 °C fridge and equilibrated for 1 h before turning the blue LED strip on (8.0 mW/cm^2). Vials were removed at 30 min, 60 min, 90 min, and 120 min and quenched by opening the vial to air. Conversion was determined by ^{1}H NMR spectroscopy using DMSO-d_6 as a solvent and tracking the disappearance of the methine peak at $\delta = 4.64$ ppm.

Example procedure for methyl acrylate small molecule analog studies

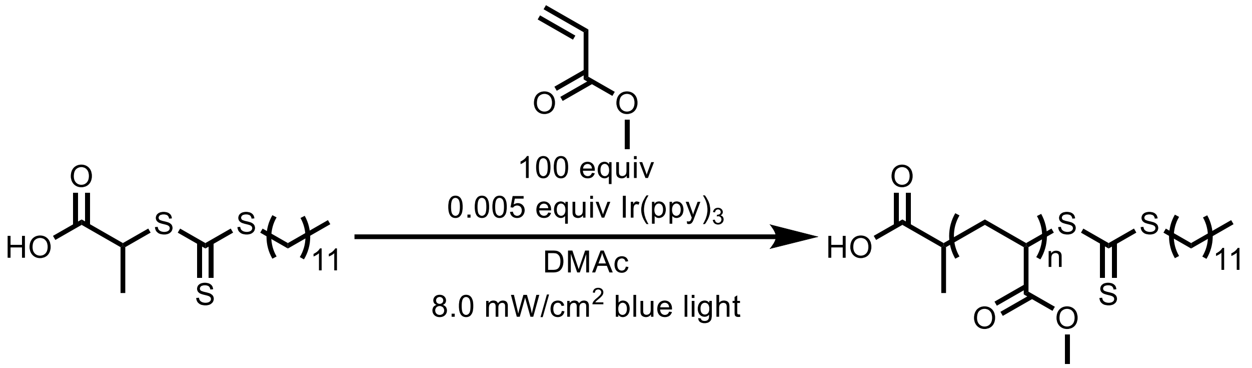

Methyl acrylate (1.02 g, 11.9 mmol), 2-(Dodecylthiocarbonothioylthio)propionic acid (41.6 mg, 1.19×10^{-1} mmol), *fac*-Ir(ppy)$_3$ (3.88×10^{-1} mg, 5.93×10^{-4} mmol) were dissolved in DMAc (1.57 mL) with DMF as an internal standard in 2-dram vials and capped with rubber septa. After degassing the solution for 5 min with Argon, the vials were placed in the 2 °C fridge and equilibrated for 1 h before turning the blue LED strip on (8.0 mW/cm^2). Vials were removed at 30 min, 60 min, 90 min, and 120 min and quenched by opening the vial to air. Conversion was determined by ^{1}H NMR spectroscopy using DMSO-d_6 as a solvent and tracking the disappearance of the methine peak at $\delta = 4.64$ ppm.

Example procedure for Single-Unit Monomer Insertion Reaction

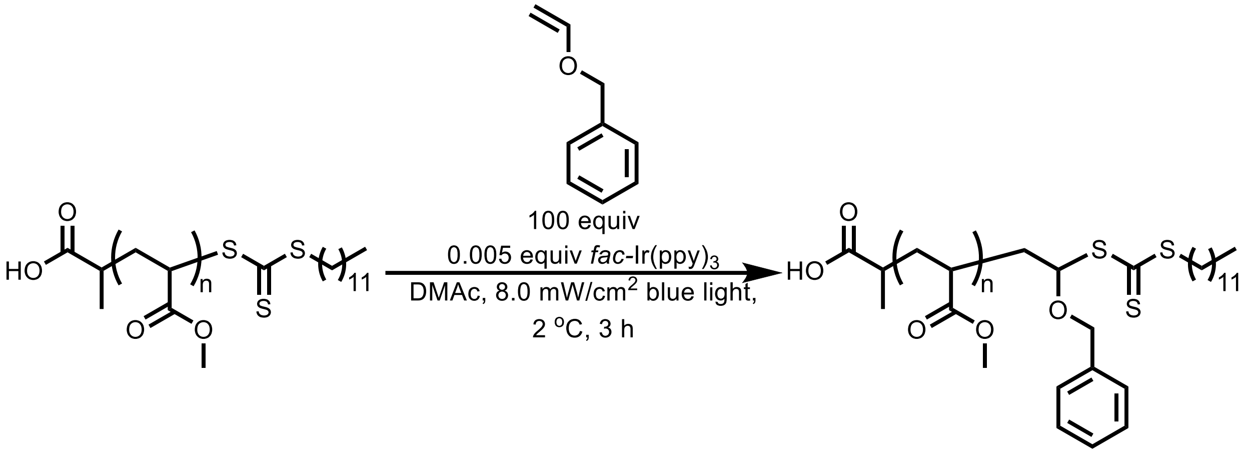

PMA (M_n = 8000 g/mol, $Đ$ = 1.06, 250 mg, 3.13 × 10^{-2} mmol), benzyl vinyl ether (419 mg, 3.13 mmol), *fac*-Ir(ppy)$_3$ (1.03 × 10^{-1} mg, 1.56 × 10^{-4} mmol) were dissolved in DMAc (0.269 mL) in a 2-dram vial and capped with a rubber septum. After degassing the solution for 5 min with Argon, the vial was placed in the 2 °C fridge and equilibrated for 1 h before turning the blue LED strip on (8.0 mW/cm^2). The sample was irradiated for 3 h and quenched by opening the vial to air. The reaction mixture was then precipitated into an excess of chilled methanol 2×, and then dried under vacuum and characterized by ^{1}H NMR spectroscopy and SEC.

Example procedure for chain extension

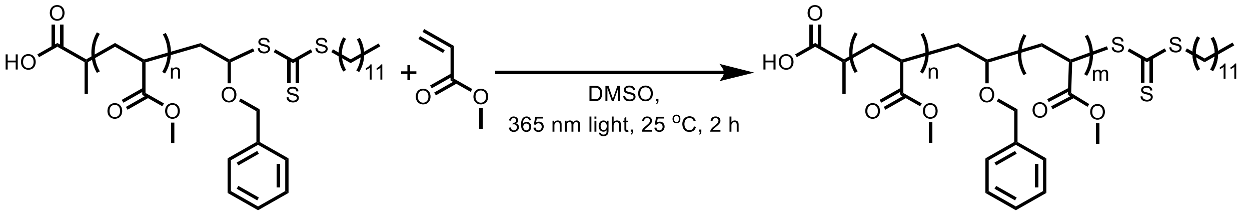

PMA-BVE (M_n = 8134 g/mol, $Đ$ = 1.10, 250 mg, 3.07 × 10^{-2} mmol) and MA (660 mg, 7.67 mmol) were dissolved in DMAc (3.14 mL) in a 2-dram vial and capped with a rubber septum. After degassing the solution for 5 min with Argon, the vial was placed approximately 4 cm from the 365 nm light source. The sample was irradiated for 2 h at 25 °C and quenched by opening the vial to air. The reaction mixture was then precipitated into an excess of chilled methanol 2×, and then dried under vacuum and characterized by ^{1}H NMR spectroscopy and SEC.

Example procedure for control SUMI studies (blue light)

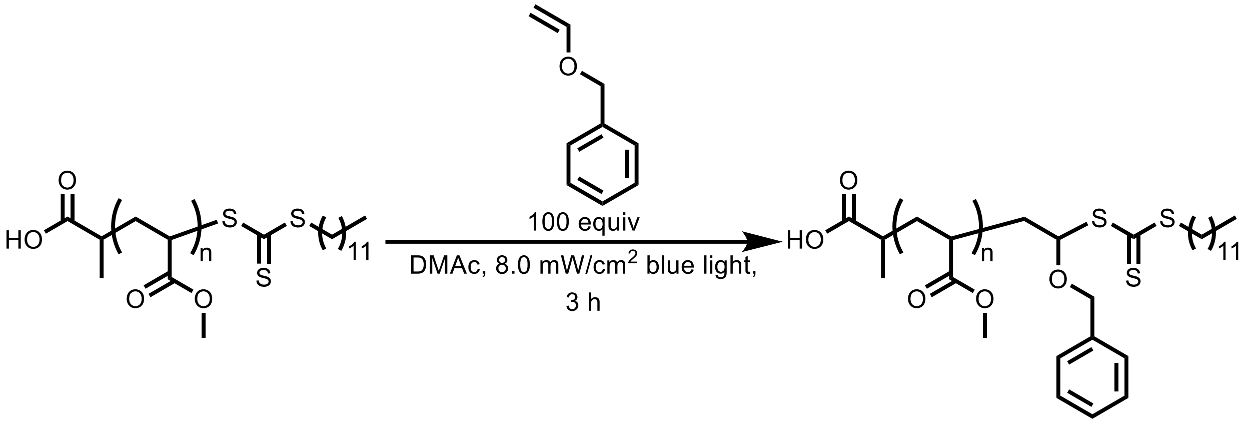

PMA (M_n = 8000 g/mol, $Đ$ = 1.06, 250 mg, 3.13 × 10^{-2} mmol) and benzyl vinyl ether (419 mg, 3.13 mmol) were dissolved in DMAc (0.279 mL) in a 2-dram vial and capped with a rubber septum. After degassing the solution for 5 min with Argon, the vial was either placed in the 2 °C fridge and equilibrated for 1 h or kept at room temperature before turning the blue LED strip on (8.0 mW/cm^2). The sample was irradiated for 3 h and quenched by opening the vial to air. The reaction mixture was then precipitated into an excess of chilled methanol 2×, and then dried under vacuum and characterized by ^{1}H NMR spectroscopy and SEC.

Example procedure for control SUMI studies (365 nm light)

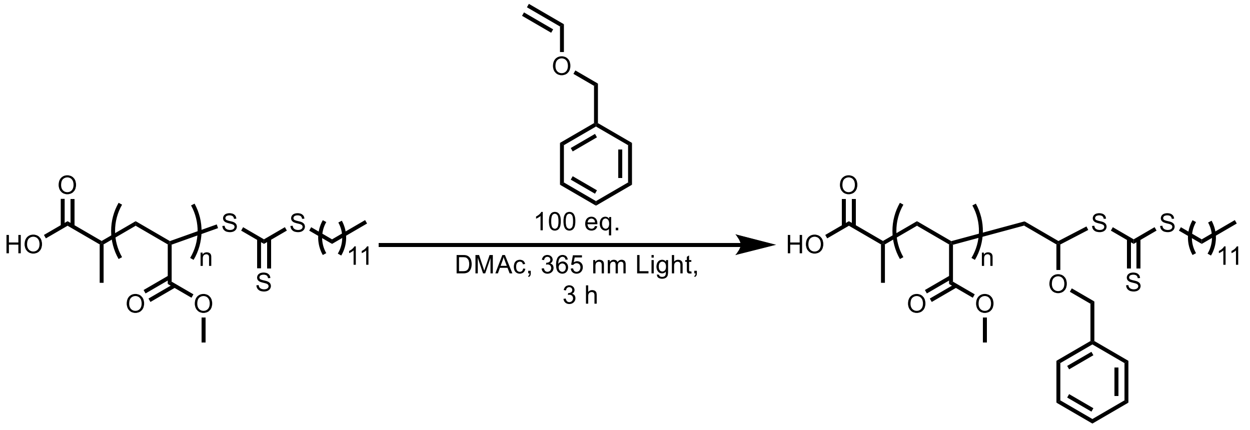

PMA (M_n = 8000 g/mol, $Đ$ = 1.06, 250 mg, 3.13 × 10^{-2} mmol) and benzyl vinyl ether (419 mg, 3.13 mmol) were dissolved in DMAc (0.279 mL) in a 2-dram vial and capped with a rubber septum. After degassing the solution for 5 min with Argon, the vial was either placed in the 2 °C fridge and equilibrated for 1 h or kept at room temperature before turning the 365 nm light on. The sample was irradiated for 3 h and quenched by opening the vial to air. The reaction mixture was then precipitated into an excess of chilled methanol 2×, and then dried under vacuum and characterized by ^{1}H NMR spectroscopy and SEC.

Additional Figures

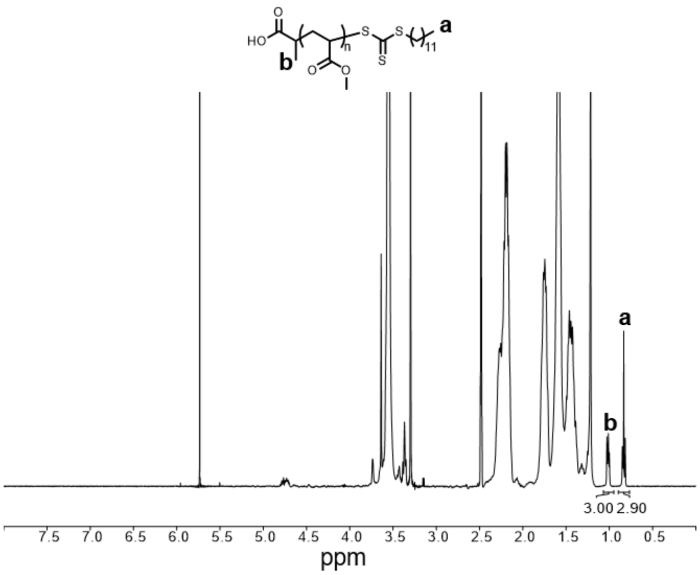

Figure S1. Starting PMA (M_n = 8000 g/mol, $Đ$ = 1.06) ^{1}H NMR spectrum for SUMI reactions.

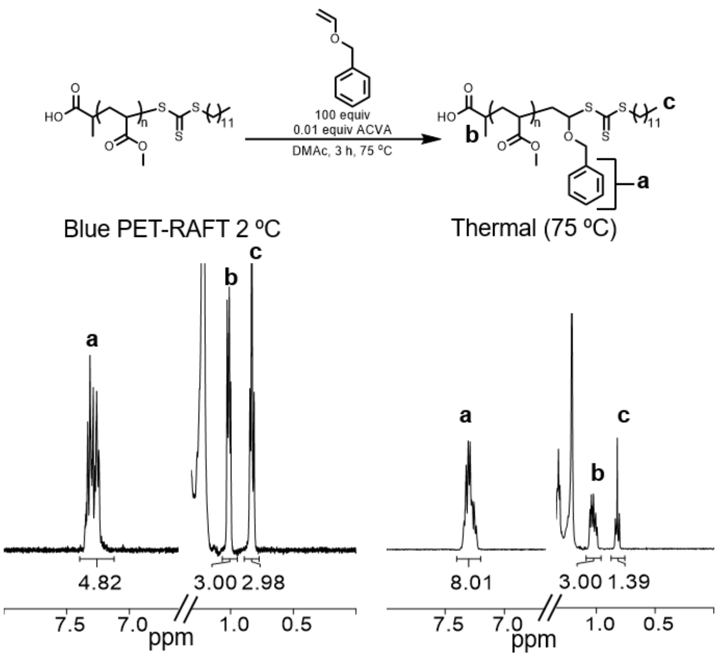

Figure S2. Comparison of PET-RAFT SUMI reactions conducted at 2 °C vs. thermal SUMI reactions conducted at 75 °C via ^{1}H NMR spectroscopy.

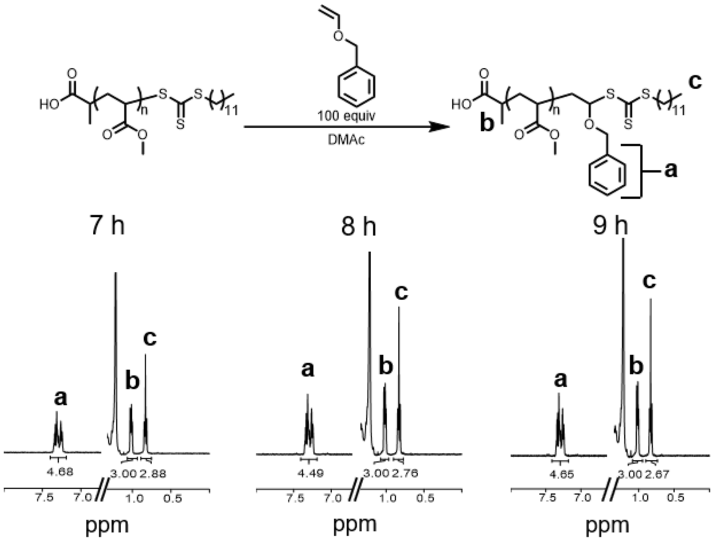

Figure S3. Comparison of SUMI reactions with increasing time at 25 ºC via ^{1}H NMR spectroscopy.

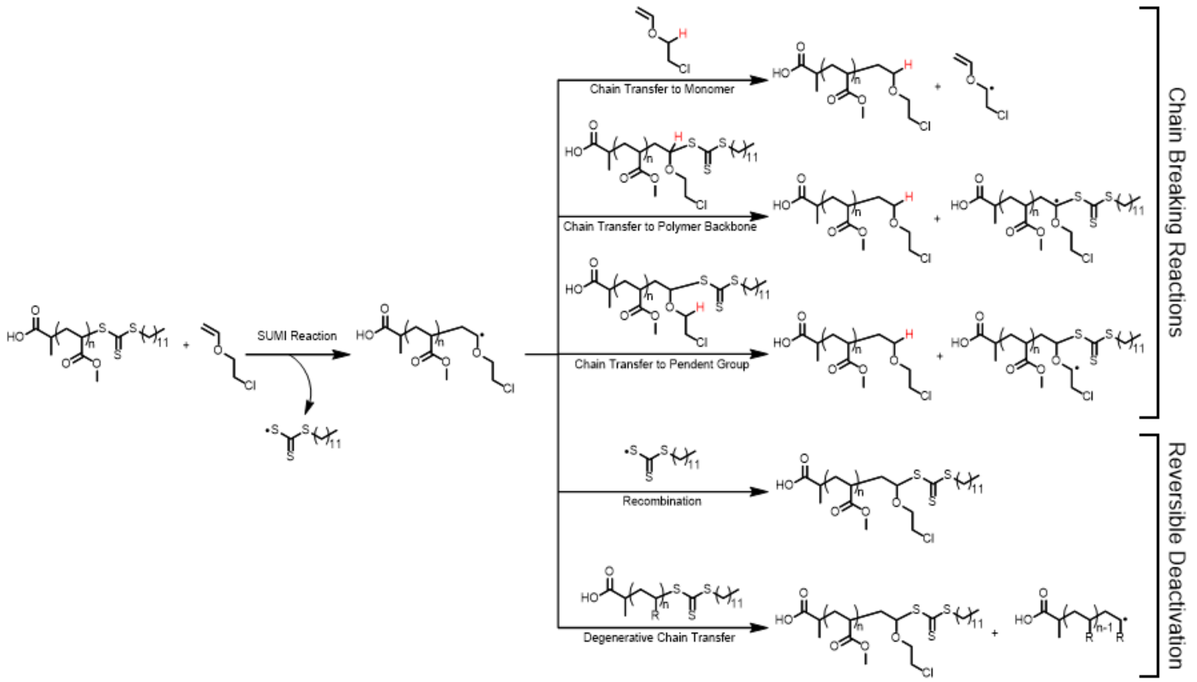

Figure S4. Possible termination and chain transfer events following the SUMI reaction. Chain breaking reaction events result in irreversibly terminated polymer chains. Reversible deactivation events result in polymers with TCT chain ends.

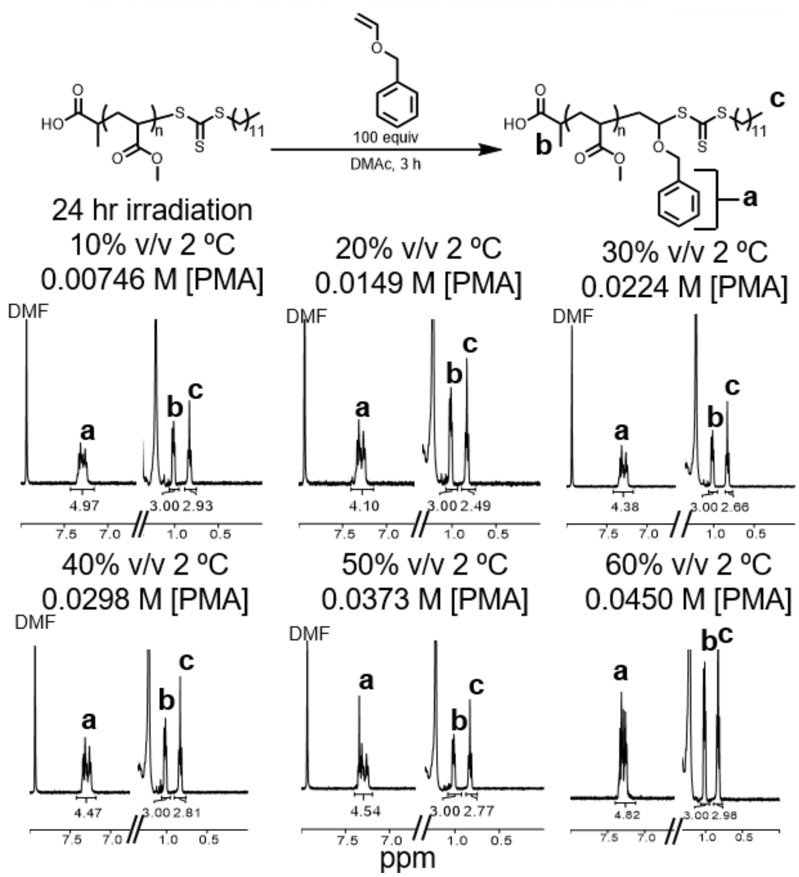

Figure S5. Comparison of SUMI reactions with increasing polymer concentrations conducted at 2 °C via ^{1}H NMR spectroscopy.

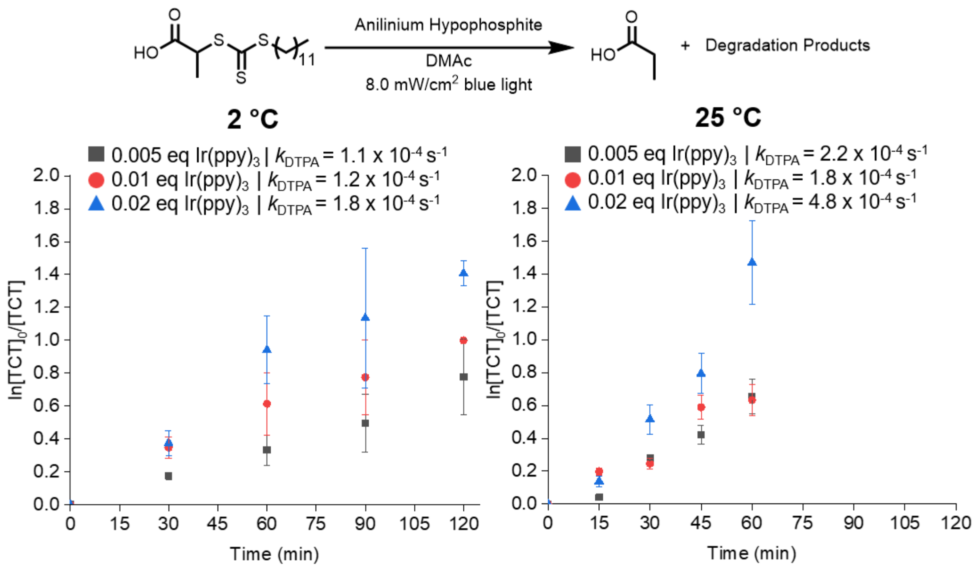

Figure S6. Trapping studies conducted at 2 °C and 25 °C with different catalyst loadings with respect to the concentration of DTPA.

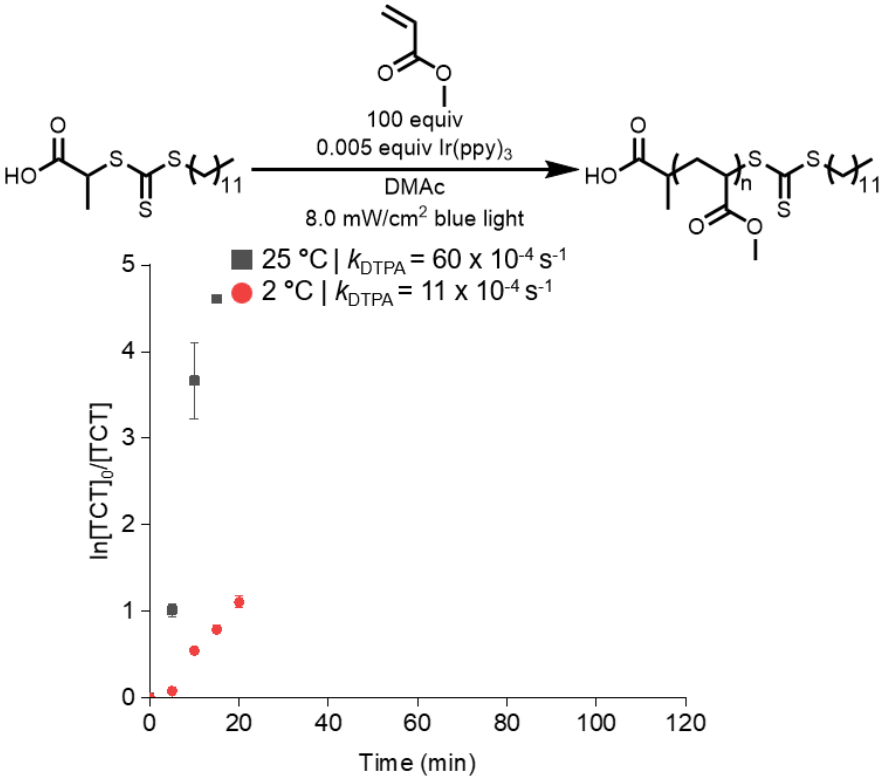

Figure S7. Small molecule studies conducted with MA at 2 °C and 25 °C with a 0.005 eq. catalyst loading with respect to the concentration of DTPA.

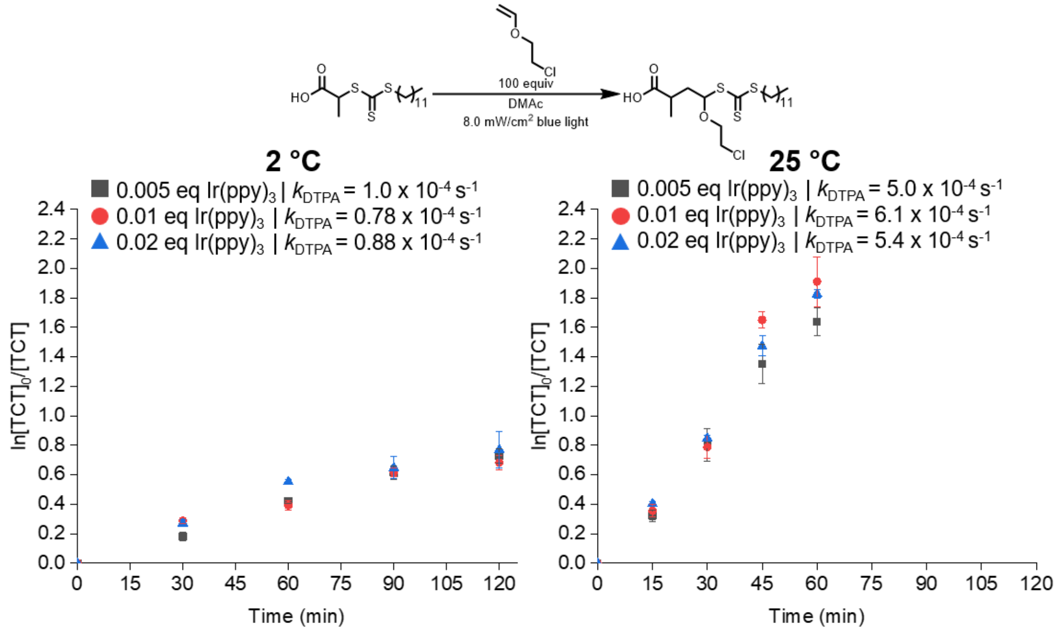

Figure S8. Small molecule studies conducted with 2-ClEVE at 2 °C and 25 °C with different catalyst loadings with respect to the concentration of DTPA.

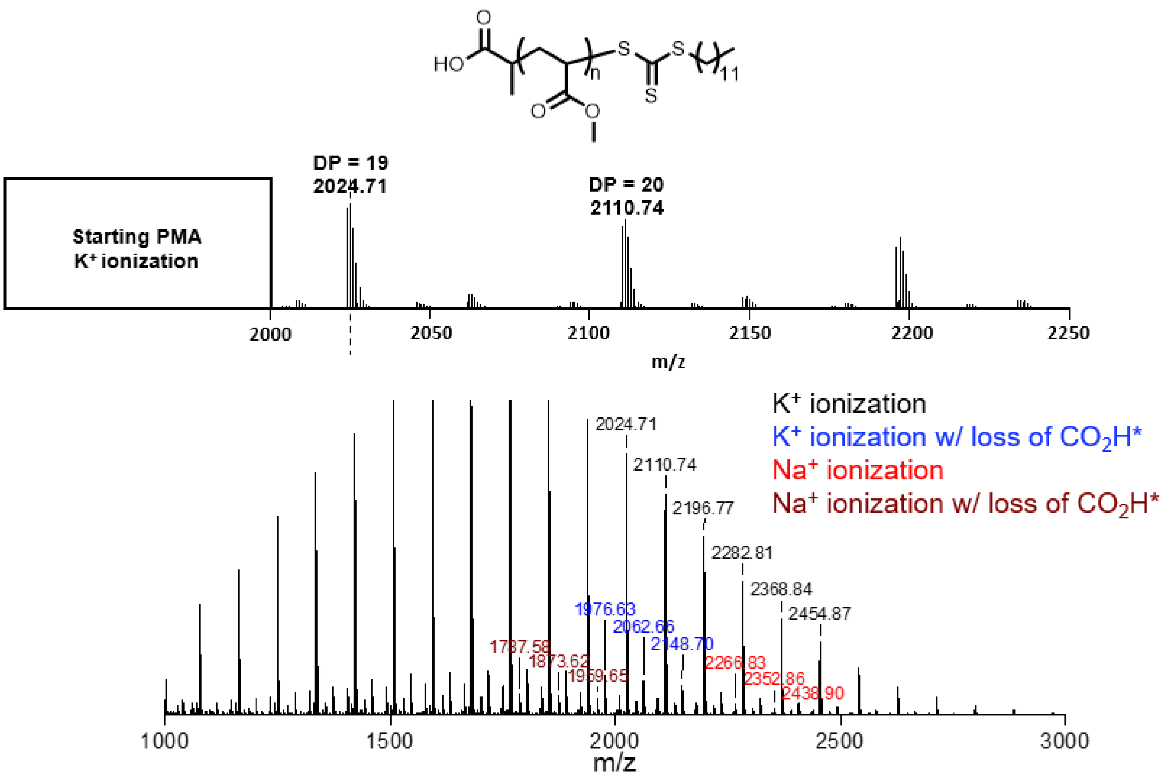

Figure S9. MALDI-TOF spectrum of starting PMA (M_n = 2,800 g/mol, $Đ$ = 1.07). *Proposed fragmentation occurring during the ionization process.[2-5]

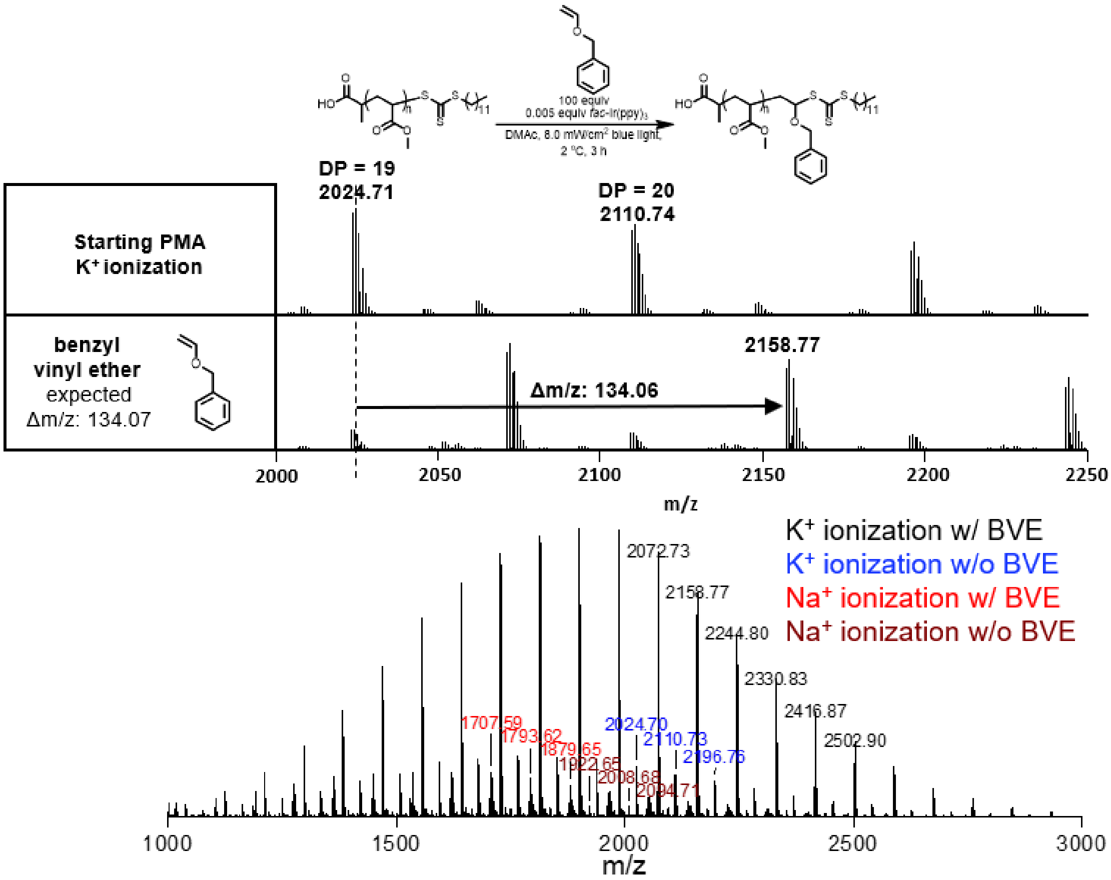

Figure S10. MALDI-TOF spectrum of PMA-BzVE.

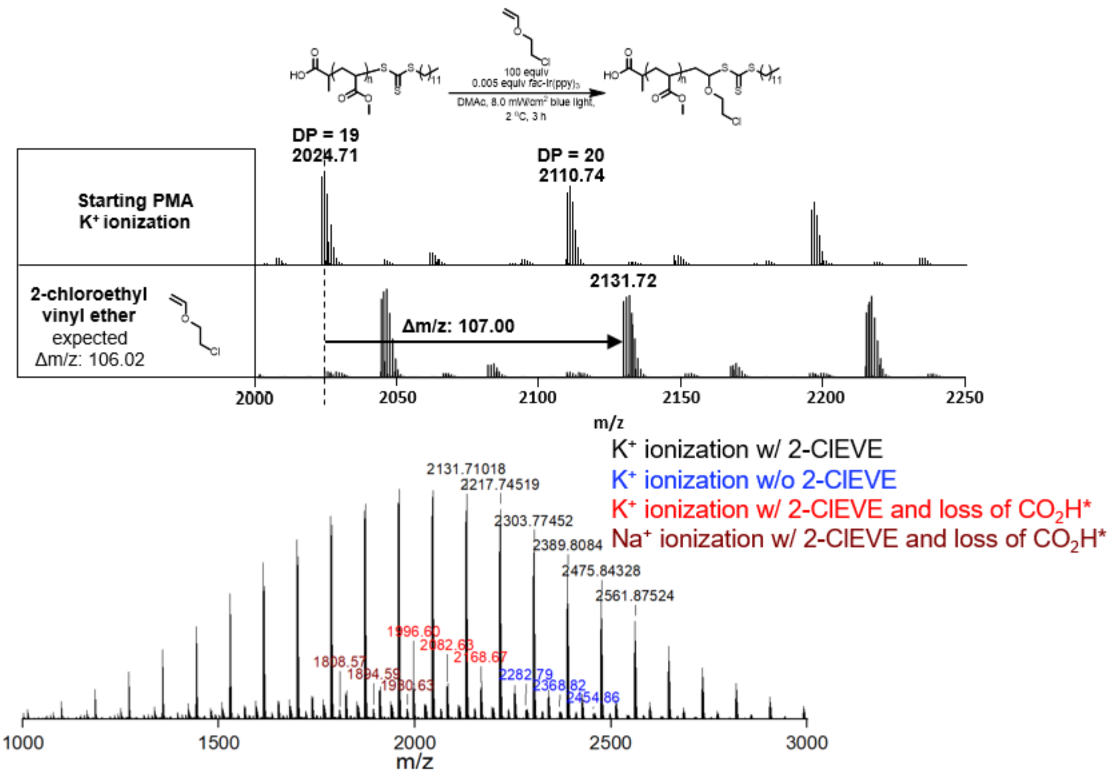

Figure S11. MALDI-TOF spectrum of PMA-(2-ClEVE). *Proposed fragmentation occurring during the ionization process.[2-5]

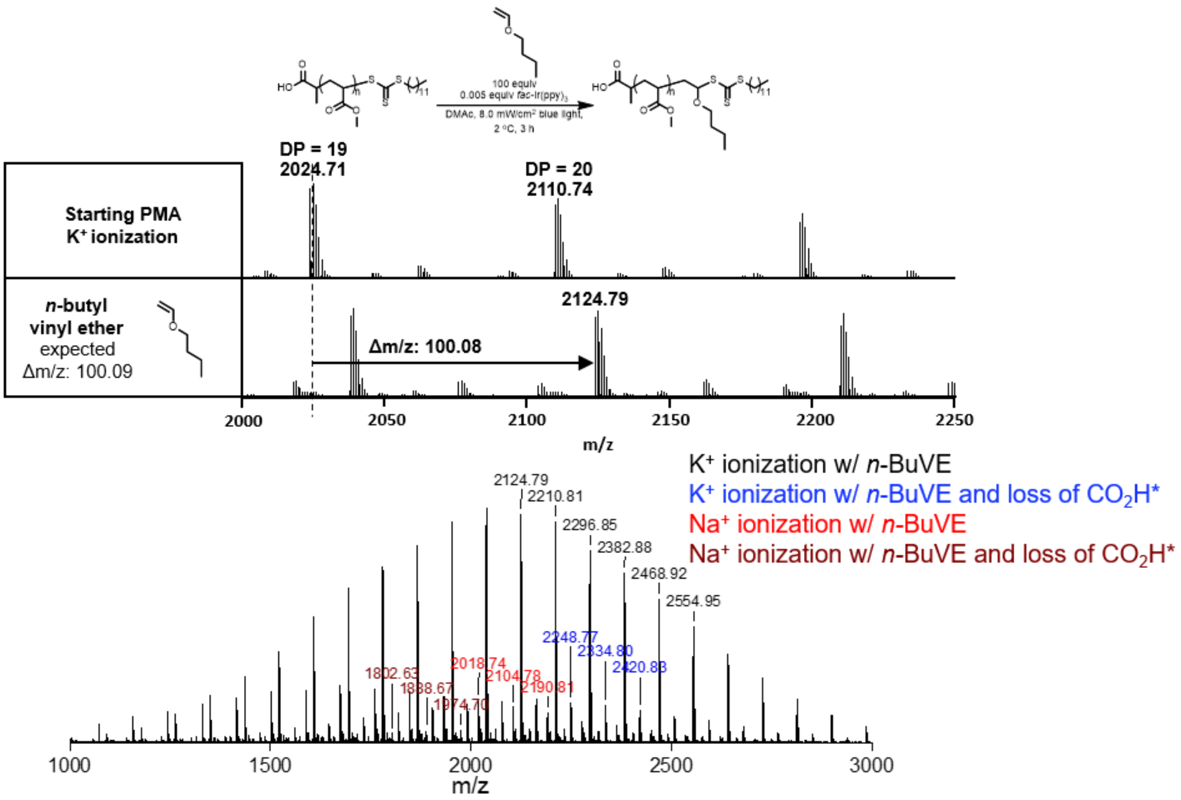

Figure S12. MALDI-TOF spectrum of PMA-(*n*-BuVE). *Proposed fragmentation occurring during the ionization process.[2-5]

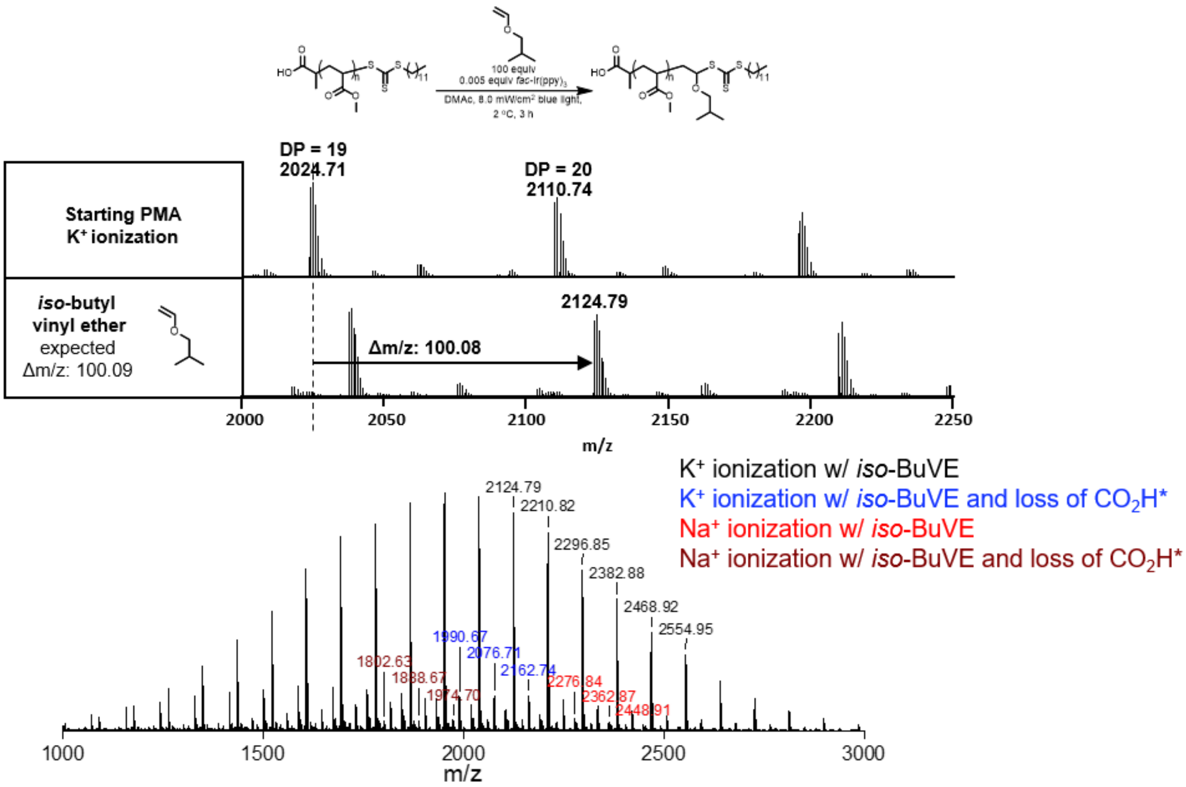

Figure S13. MALDI-TOF spectrum of PMA-(*iso*-BuVE). *Proposed fragmentation occurring during the ionization process.[2-5]

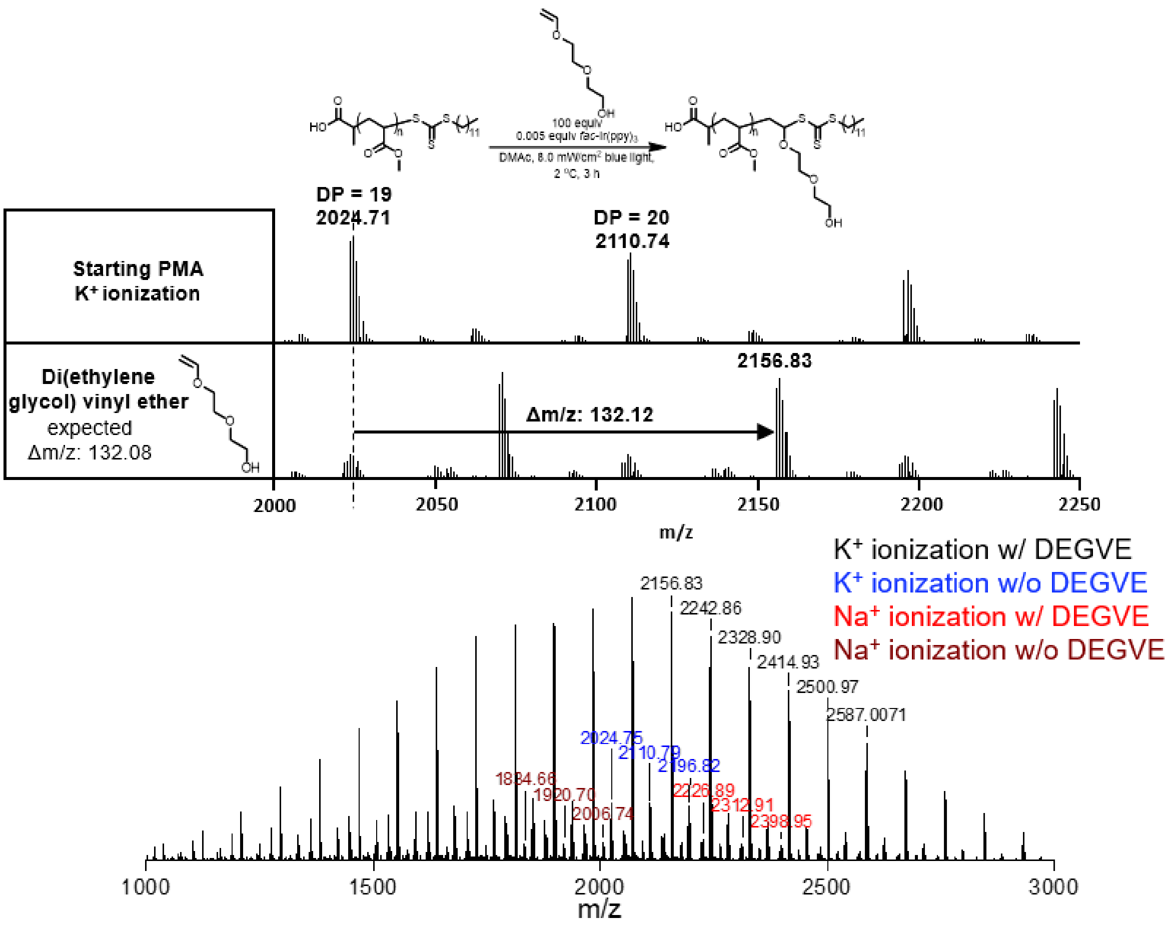

Figure S14. MALDI-TOF spectrum of PMA-DEGVE.

A.

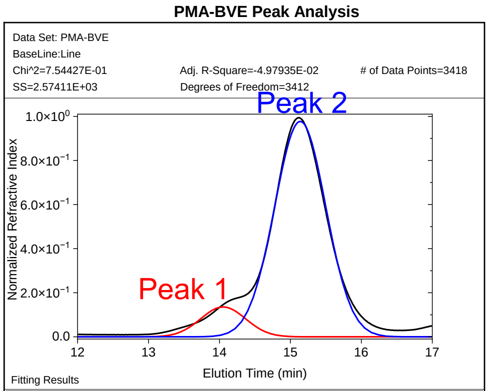

Fitting Results

Peak Index	Peak Type	Area Intg	FWHM	Max Height	Center Grvty	Area IntgP
1	Gaussian	0.1071	0.74283	0.13545	14.05181	10.84884
2	Gaussian	0.8801	0.84645	0.97678	15.13849	89.15116

Results

Peak Results

	**Peak 1**	**Peak 2**
**General (mL/(mg cm))**		
**UV Ext. Coef. (mL/(mg cm))**	0.013	0.011
**Masses**		
**Injected Mass (µg)**	1014.00	1014.00
**Calculated Mass (µg)**	166.94	1220.68
**Mass Recovery (%)**	16.5	120.4
**Molar mass moments (g/mol)**		
**Mn**	1.254×10^4 (±7.307%)	6.895×10^3 (±6.267%)
**Mw**	1.347×10^4 (±8.382%)	7.082×10^3 (±7.089%)
**Polydispersity**		
**Mw/Mn**	1.074 (±11.120%)	1.027 (±9.462%)

B.

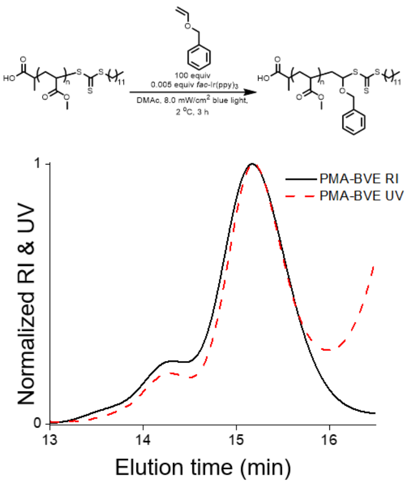

Figure S15. A. Peak analysis of PMA-BVE peaks using deconvolution to assess percentages using Origin Pro and molecular weights of lower elution time species using Astra. Since peak 1 is 2× the molecular weight of peak 2, the area integration was divided by 2 to determine the relative mole percentages of each peak. B. Normalized RI and UV traces are shown for PMA-BVE. The high molecular weight shoulder following the SUMI reaction shows UV absorbance at 365 nm, the wavelength associated with TCTs. This UV absorbance indicates that the chains are not terminated through a conventional chain-chain coupling termination pathway, in which no TCT absorbance would be observed. Instead, the remaining UV absorbance likely stems from chain breaking events either to backbone or vinyl ether pendent groups, which is followed by a polymer-polymer coupling.

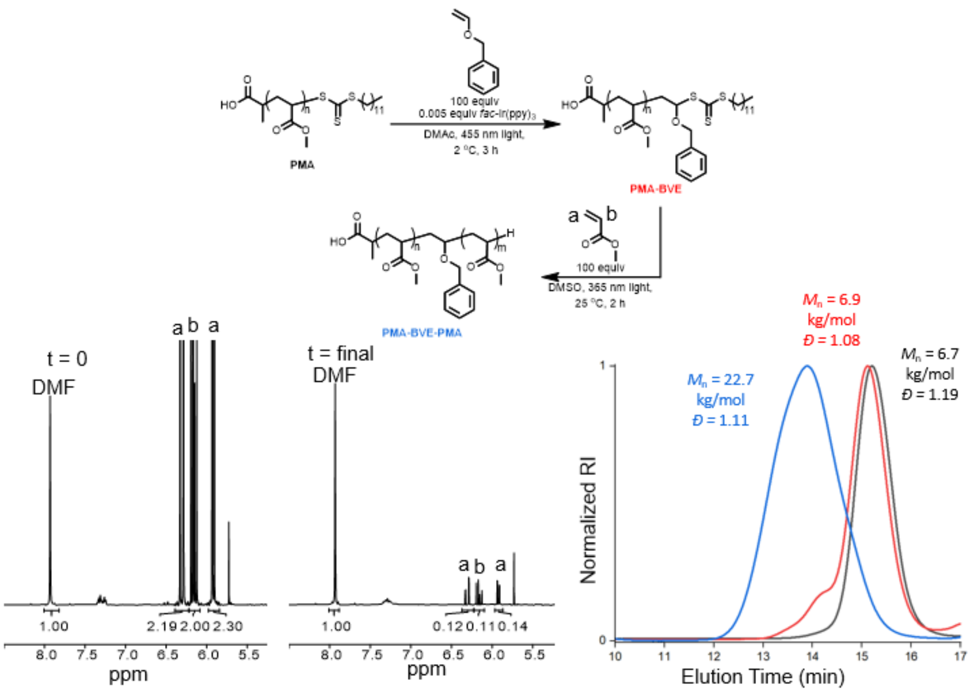

Figure S16. Chain extension of PMA-BVE with 100 equiv MA with t = 0 and final ^{1}H NMR spectra and SEC chromatograms.

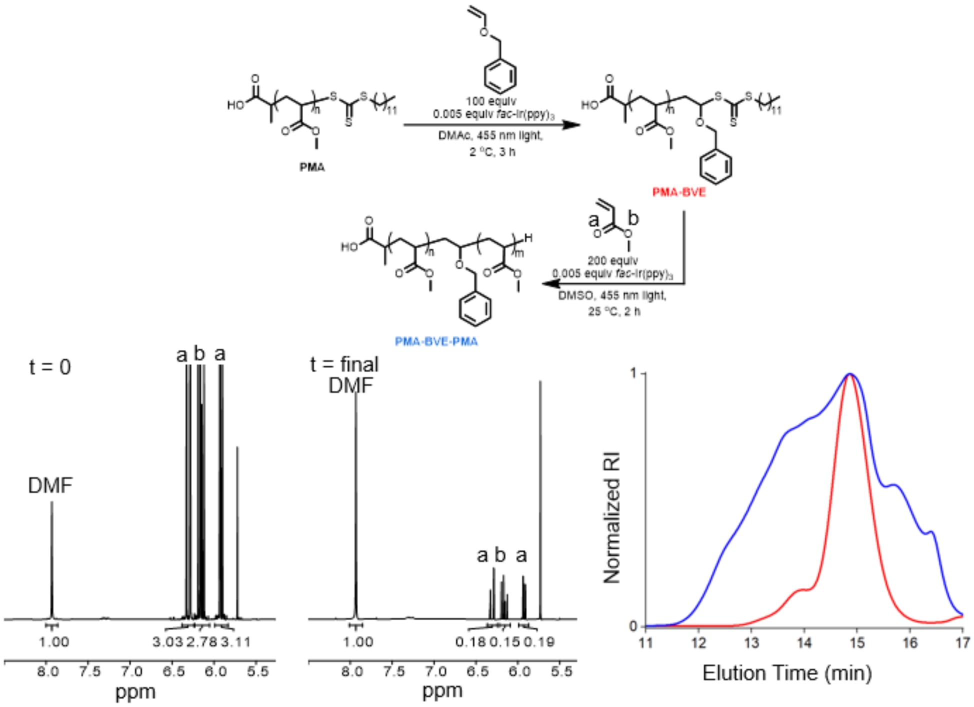

Figure S17. Chain extension using 0.005 equiv Ir(ppy)$_3$ irradiated with 455 nm light of PMA-BVE with 200 equiv MA with t = 0 and final ^{1}H NMR spectra and SEC chromatograms.

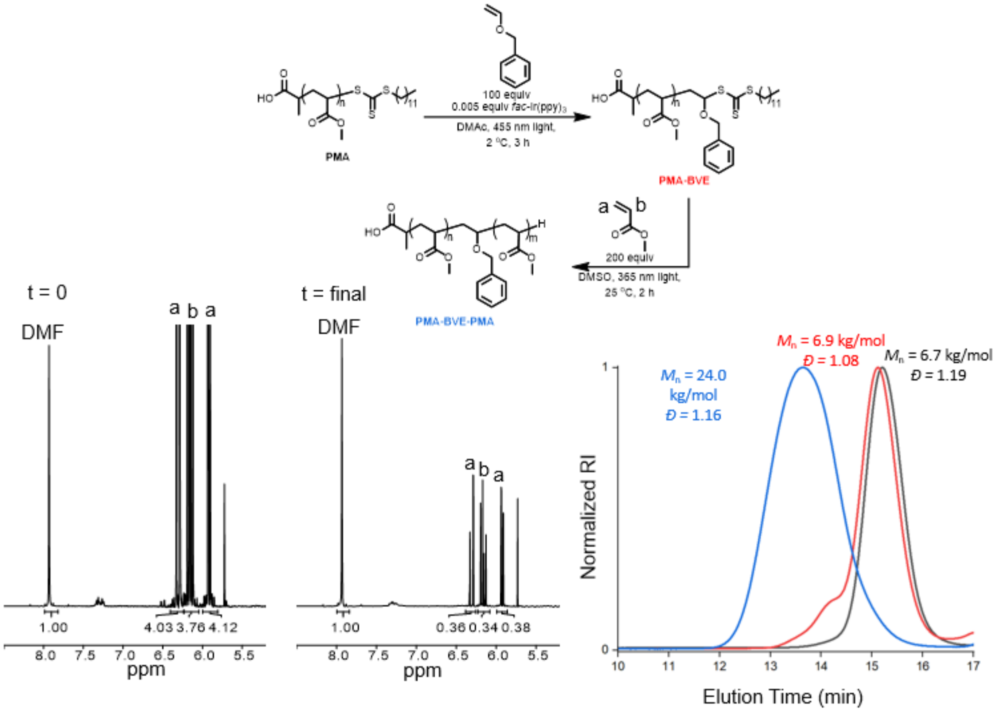

Figure S18. Chain extension of PMA-BVE with 200 equiv MA with t = 0 and final ^{1}H NMR spectra and SEC chromatograms.

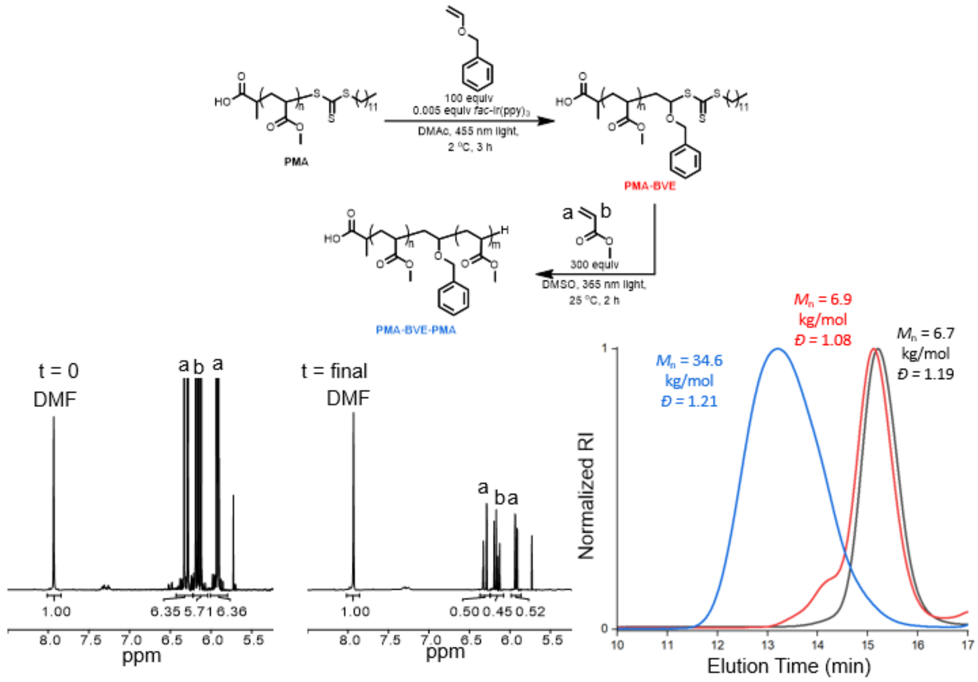

Figure S19. Chain extension of PMA-BVE with 300 equiv MA with t = 0 and final ^{1}H NMR spectra and SEC chromatograms.

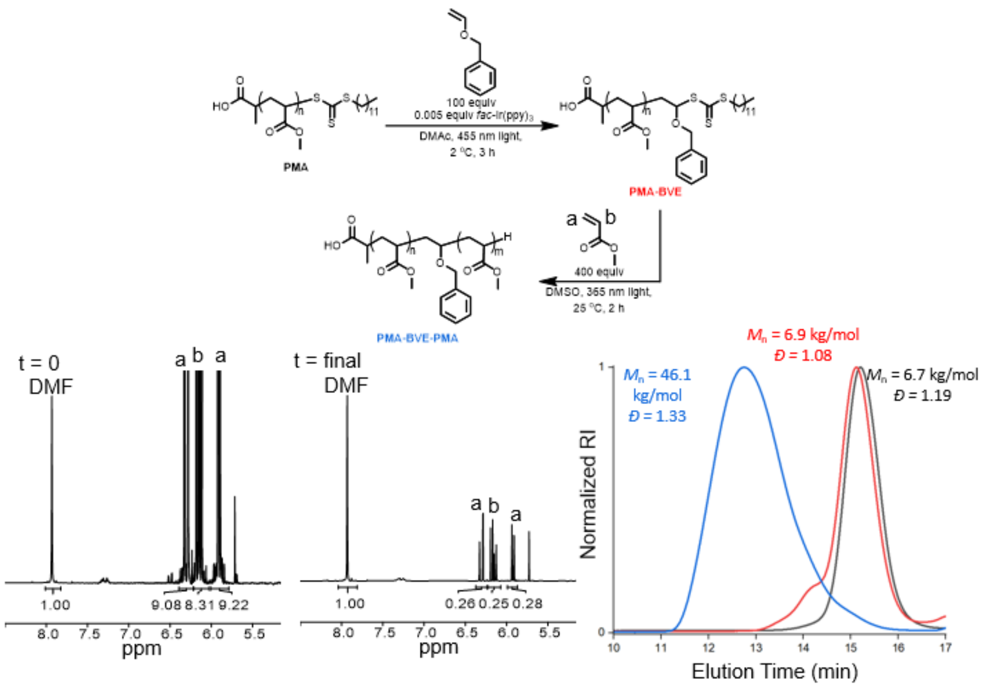

Figure S20. Chain extension of PMA-BVE with 400 equiv MA with t = 0 and final ^{1}H NMR spectra and SEC chromatograms.

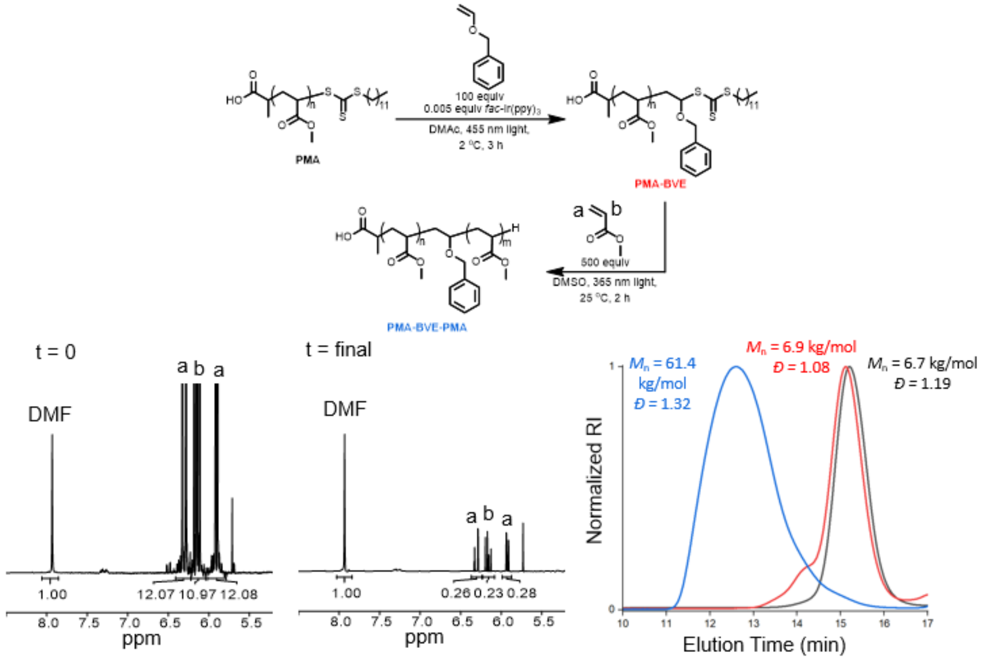

Figure S21. Chain extension of PMA-BVE with 500 equiv MA with t = 0 and final ^{1}H NMR spectra and SEC chromatograms.

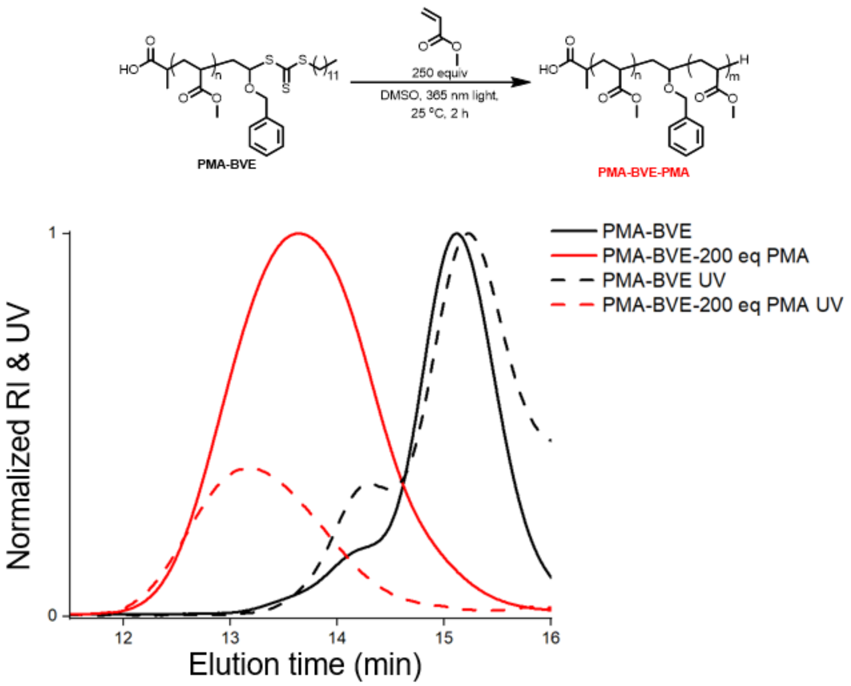

Figure S22. Normalized RI and UV traces for PMA-BVE and PMA-BVE-PMA. Following the chain extension from PMA-BVE there is a large decrease in UV indicating large amounts of irreversible termination events occur during the photoiniferter chain extension.

References:

1. Skey, J.; O'Reilly, R. K. Facile one pot synthesis of a range of reversible addition–fragmentation chain transfer (RAFT) agents. *Chem. Commun.* **2008**, 4183–4185.
2. Wan, D.; Zhou, Q.; Pu, H.; Yang, G. Controlled Radical Polymerization of *N* -vinylcaprolactam Mediated by Xanthate or Dithiocarbamate. *J. Polym. Sci. A Polym. Chem.* **2008**, *46*, 3756–3765.
3. Peacock, P. M.; McEwen, C. N. Mass Spectrometry of Synthetic Polymers. *Anal. Chem.* **2004**, *76*, 3417–3428.
4. Gies, A. P.; Hercules, D. M.; Ellison, S. T.; Nonidez, W. K. MALDI-TOF MS Study of Poly(*p* -Phenylene Terephthalamide) Fibers. *Macromolecules* **2006**, *39*, 941–947.
5. Montaudo, G.; Samperi, F.; Montaudo, M. S. Characterization of Synthetic Polymers by MALDI-MS. *Prog. Polym. Sci.* **2006,** *31*, 277–357.
